# Mycobiomes of Young Beech Trees Are Distinguished by Organ Rather Than by Habitat, and Community Analyses Suggest Competitive Interactions Among Twig Fungi

**DOI:** 10.3389/fmicb.2021.646302

**Published:** 2021-04-15

**Authors:** Abu Bakar Siddique, Paolo Biella, Martin Unterseher, Benedicte Riber Albrectsen

**Affiliations:** ^1^Department of Ecology and Environmental Sciences, Faculty of Science and Technology, Umeå University, Umeå, Sweden; ^2^Department of Biotechnology and Biosciences, University of Milano-Bicocca, Milan, Italy; ^3^Montessori-Schule, Greifswald, Germany; ^4^Department of Plant Physiology, Umeå Plant Science Centre, Umeå University, Umeå, Sweden

**Keywords:** *Fungal endophytes*, Illumina sequencing, plant organ, colonization, diversity, abundance, guild analyses, network analysis

## Abstract

Beech trees (*Fagus sylvatica*) are prominent keystone species of great economic and environmental value for central Europe, hosting a diverse mycobiome. The composition of endophyte communities may depend on tree health, plant organ or tissue, and growth habitat. To evaluate mycobiome communalities at local scales, buds, and twigs were sampled from two young healthy mountain beech stands in Bavaria, Germany, four kilometers apart. With Illumina high-throughput sequencing, we found 113 fungal taxa from 0.7 million high-quality reads that mainly consisted of Ascomycota (52%) and Basidiomycota (26%) taxa. Significant correlations between richness and diversity indices were observed (*p* < 0.05), and mycobiomes did not differ between habitats in the current study. Species richness and diversity were higher in twigs compared to spring buds, and the assemblages in twigs shared most similarities. Interaction network analyses revealed that twig-bound fungi shared similar numbers of (interaction) links with others, dominated by negative co-occurrences, suggesting that competitive exclusion may be the predominant ecological interaction in the highly connected twig mycobiome. Combining community and network analyses strengthened the evidence that plant organs may filter endophytic communities directly through colonization access and indirectly by facilitating competitive interactions between the fungi.

## Introduction

Fungal endophytic communities are shaped by numerous factors internally, related to the plant host, and externally, related to the growing site. Internal factors may include host plant species and genotype (Unterseher et al., 2007, 2012; Peršoh et al., 2010; Cordier et al., 2012; Bálint et al., 2013; Peršoh, 2013; Weig et al., 2013; Albrectsen et al., 2018); plant organ, e.g., leaves, twigs, and stems (Sieber and Hugentobler, 1987; Petrini, 1991; Sahashi et al., 1999; Santamaría and Diez, 2005); plant condition (Agostinelli et al., 2018); and plant development (Jumpponen and Jones, 2010; Vořıšková and Baldrian, 2013; Baldrian, 2017). External factors may include structure and diversity of the surrounding vegetation (Helander et al., 2007), antagonists such as herbivores (Albrectsen et al., 2018), local climate conditions (Hashizume et al., 2008; Cordier et al., 2012; Eusemann et al., 2016; Würth et al., 2019), geographical location (U’Ren et al., 2012), and seasonality (Karlsson et al., 2020). The complexity of the mycobiome is thus well established, although ecological insights have mostly been founded on cultivation-based techniques that tend to underestimate diversity (Siddique et al., 2017). Indeed, next-generation sequencing studies of beech suggest that any aerial organ is colonized by rich endophyte communities (Unterseher et al., 2013, 2016). However, it remains largely unexplored how colonization takes place and ideas about what kind of interactions govern the endophytic mycobiome are also, so far, highly speculative.

Organisms that share an environment form ecological networks with characteristic features of the biotic interactions that shape the network (Montesinos-Navarro et al., 2012; Biella et al., 2017; Bennett et al., 2019). The network structure can, therefore, indicate strategies of resource use and provide evidence of direct and indirect competition between members of the network, thus providing a tool to understand species assemblages and the processes that led to them (Sauve et al., 2014; Chagnon et al., 2016), including substrate filtering (e.g., organ or tissue dependency). Although we have evidence that genotype (Albrectsen et al., 2018) and tree health (Agostinelli et al., 2018) shape the mycobiome, fungal participants will also continuously interact with each other within the fungal network. Thus, in network analysis, we may distinguish between two interaction layers: (1) interactions between host and fungi and (2) interactions among the fungi (Toju et al., 2015; Guerreiro et al., 2018; Cobo-Díaz et al., 2019). The plant–fungi layer can further provide insights into the importance of plant internal factors, such as plant organ type, and external factors, such as growing condition.

Several studies investigated plant–fungi interactions. Ectomycorrhizal fungi exhibit a high specificity with respect to their interactions with plant roots, meaning that each mycorrhizal species inoculates only a few plant species (Tedersoo et al., 2008; Toju et al., 2014). In contrast, studies of root–endophytic fungi suggest less specificity, indicating a generalist strategy employed by these fungi that inhabit and interact with a broad range of host plant taxa (Walker et al., 2011; Knapp et al., 2012). Growth conditions define plant growth potential, and it has also been suggested that mycobiomes in forest trees may depend on site conditions (Cordier et al., 2012; U’Ren et al., 2012; Siddique et al., 2017). The mycobiome structure has been studied in twigs of several forest trees including *Fagus sylvatica* (Sieber and Hugentobler, 1987; Danti et al., 2002), *Quercus cerris* (Ragazzi et al., 2003), *Fraxinus excelsior* (Bakys et al., 2009; Chen, 2012), *Betula pubescens* (Barengo et al., 2000), *Populus* spp. (Martín-García et al., 2011), *Pinus* sp. (Botella et al., 2010; Sanz-Ros et al., 2015; Bußkamp et al., 2020), *Abies concolor*, and *Picea abies* (Sieber, 1989). Twig mycobiomes associate with both biotic and abiotic factors, e.g., air quality (Bakys et al., 2009), geography and seasonality (Göre and Bucak, 2007), genotype and growth (Lamit et al., 2014; Sanz-Ros et al., 2015), and plant health (Sieber, 1989; Ragazzi et al., 2003). However, few studies have surveyed endophytes in buds (Johnson and Whitney, 1992; Pirttilä et al., 2003; Chen, 2012).

In general, we have little knowledge about plants’ ability to filter endophytic fungi, for example, through organ-specific properties that may either directly influence the colonization or establishment of a certain fungal taxon or indirectly affect fungal taxa by shaping interactions between community members. It has, for example, been suggested that undeveloped organs are endophyte-free (Toti et al., 1993) which was opposed by endophytes colonizing pine buds (Pirttilä et al., 2003). Cold-season grass endophytes are systemic and transferred via seeds between generations (Clay and Schardl, 2002). The variation between the network structures of different plant parts may shed light on otherwise hidden features that shape endophytic communities (Parrish and Bazzaz, 1979; Albrecht et al., 2010). Specifically, we hypothesize that in a plant organ colonized by few fungal species, the plant–fungi network will be characterized by a few interaction links, indicating that random processes determine fungal colonization, like the processes observed in interactions established by plants of early successional stages (Albrecht et al., 2010). Conversely, when plant organs host a high diversity of fungi, the plant–fungi network will be more complex and more structured because, as studies from other network types reveal, a diverse community supports a high number of interactions between taxa (Albrecht et al., 2010). In addition, we also expect to find that a higher taxa diversity will increase the competition between interacting species, as shown, for example, in plant networks with pollinators (Parrish and Bazzaz, 1979).

Considering the effect of local habitat conditions in previous studies, we hypothesize that plant organs may filter fungal communities differently, potentially overlaid by differences in growth habitat. With the use of Illumina sequencing techniques, we studied the mycobiome of twigs and buds of young European beech trees from two forest stands. We employed multivariate techniques and diversity index algorithms to determine endophyte community characteristics such as taxon representation, ecological guild membership, and diversity properties grouped on the basis of organ and site. Network analyses further evaluated the way that the plant and fungi interacted with each other, by describing patterns due to plant organ type and to the facilitative or competitive relationships between endophytic fungal taxa.

## Materials and Methods

### Sampling Design and Field Work

**FIGURE 1:**
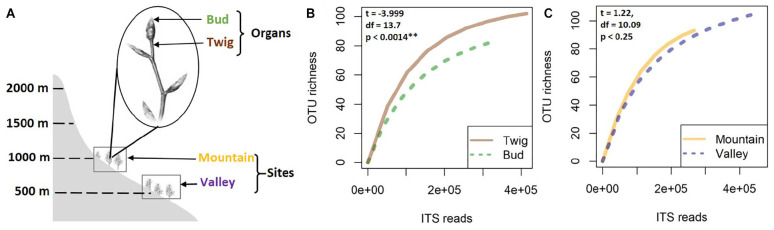
For this study, samples of buds and twigs (the distal internode on a branch) were collected from 4-year-old beech trees at two study sites in Bavaria **(A)**. Species accumulation curves of fungal mycobiomes were analyzed according to organ **(B)** and site **(C)**. Higher species richness was indicated for twigs compared to buds, whereas only nonsignificant differences separated the sites.

This study was part of a larger research project seeking to enhance knowledge of mycobiome dynamics of European beech (*F. sylvatica* L.) in different environmental conditions. Here, buds and twigs were sampled from experimentally introduced trees (Unterseher et al., 2016) at two different altitudes (sites) in a beech-dominated forest of the mountain massif “Untersberg” in the Berchtesgaden Alps, Bavaria, Germany. The two sites (referred to as “Valley” and “Mountain”) were at 517 and 975 masl, respectively, both on slopes with similar aspects, with the same soil type (Leptosol over limestone) and similar surrounding plant species (e.g., *Acer pseudoplatanus*, *P. abies*, *Daphne mezereum*, *Dentaria enneaphyllos*, *Helleborus niger*, and *Hepatica nobilis*) (Siddique and Unterseher, 2016). Sampling took place in April 2015. Five 4-year-old trees per site were randomly selected for the present study. From each tree, 10 twigs with unopened buds were sampled (Figure 1A). From each branch, only the terminal bud and the associated twig region were collected, pooled by organ, and kept cool (4°C). In total, 20 samples were obtained, brought to the lab in Greifswald within 1 day for surface sterilization. In brief, samples were sterilized by placing briefly in sterile distilled water, in 70% EtOH for 2 min, in 1% NaHClO for 5 min, and in 70% EtOH for 1 min and rinsing in sterile distilled water (Schulz et al., 1993; Unterseher and Schnittler, 2009). Samples were stored at −80°C until further processing.

### DNA Extraction, ITS Library Preparation, and Illumina Sequencing

Buds and twig samples were thawed and homogenized for 1 min with sterile-distilled water in a commercial blender and filtered through three analytical sieves (630, 200, and 100 μm) in order to obtain differently sized particles as outlined at the first part of “particle filtration” in Unterseher and Schnittler (2009). Buds generated substantial quantities of foam during sample processing. This protocol was originally for plant leaves, but we adopted it for buds and twigs samples in order to synchronize with the previous project plans (refer to Siddique and Unterseher, 2016; Unterseher et al., 2016). Approximately 100 mg (fresh weight) of the 100–200-μm-sized retained buds and twig samples was used for DNA extraction. Genomic DNA was extracted from the 20 samples with a Charge Switch gDNA Plant Kit (Invitrogen) according to the manufacturer’s instructions. An ITS (internal transcribed spacer) Illumina amplicon library was prepared with two consecutive PCR steps to obtain sample-specific tags and fungal-specific ITS1F and ITS4 primers (“Supplementary File 1,” according to Siddique and Unterseher, 2016; Unterseher et al., 2016). The Illumina ITS library was sequenced as a 300-bp paired-end sequence on an Illumina MiSeq platform (Illumina Inc.) according to the protocols of the Genetics Section, Biocenter of the LMU Munich.

### Data Processing Workflow

The raw Illumina data (SRA accessions: SRX9767994, SRX9767995, and SRX9767996) were demultiplexed and filtered for high-quality reads (for details see “Supplementary File 1”) in QIIME (Navas-Molina et al., 2013; Eusemann et al., 2016; Unterseher et al., 2016). Extraction of ITS1 reads (forward R1 Illumina reads) was conducted with ITSx (Bengtsson-Palme et al., 2013) followed by reference-based chimera checking (Nilsson et al., 2015) and open-reference operational taxonomic unit (OTU) picking with USEARCH (complete-linkage clustering) at 97% similarity (Rideout et al., 2014). Selection of representative sequences (sh_refs_qiime_ver6_dynamic_s_09.02.2014, refer to Supplementary File 1: Qiime commands) and taxonomy assignment were achieved using the database sh_qiime_release_s_09 (Kõljalg et al., 2013). Final quality filtering of OTUs involved the removal of unique (occurring in only one sample) and rare OTUs (having less than ten reads, cf. Brown et al., 2015). As unassigned OTUs can significantly contribute to the level of diversity (Zamkovaya et al., 2020), we decided to not to exclude them from the analyses. Thus, unassigned OTUs that were not assigned at the fine taxonomical level (i.e., reliably assigned only at order or class or phylum or kingdom) were clustered into reliable pseudo-species using the LULU algorithm (Froslev et al., 2017) with the original script for R provided by the author of the algorithm in R v 3.6^1^. The pseudo-species thus obtained were added to the table of the assigned OTUs LULU_OTU_Table_Spec, Supplementary File 2. After this, we created matrices of plant samples by fungal taxa (assigned OTUs at low taxonomic level and pseudo-species), upon which the following analyses were based.

### Biodiversity Analysis and Functional Guild Assessment

Biodiversity of bud- and twig-inhabiting endophytes was calculated in terms of fungal taxon richness, accumulation curves (also known as rarefaction curves), Fisher’s alpha (richness index: a count of species), Shannon index (considering both richness and abundance), and the three Hill numbers from Hill’s series (Hill, 1973) of diversity, which divide communities according to abundances (i.e., N1 = richness, N2 = exponent of Shannon index, abundant spp., and N3 = inverse Simpson index very abundant spp.). The similarity between the bud and twig mycobiome communities was tested with a permutational ANOSIM analysis (999 permutations on the presence–absence dataset, with Jaccard similarity, with the *vegan* library in R). Statistical tests of biodiversity differences for these parameters were undertaken with ANOVA of multivariate generalized linear models. Analyses of diversity and community composition were repeated using a rarefied data set, downsampled to the lowest number of reads per sample (7766 reads). Results are shown in Supplementary Figure 6 and Supplementary Table 1. Community composition was further analyzed and visualized by use of nonmetric multidimensional scaling (NMDS) based on Bray–Curtis dissimilarities of square root-transformed relative read abundances at the taxon level (Bray and Curtis, 1957). The distinctiveness of fungal assemblages was statistically tested with a permutational multivariate analysis of variance using Bray–Curtis distance matrices [PERMANOVA/adonis (Oksanen, 2011)]. Taxonomic compositions were visualized based on relative abundances. Most abundant orders and genera were displayed with heatmaps (see Supplementary Figure 5) based on Bray–Curtis matrices. Analyses were executed in RStudio [R version 3.6.3 (February 19, 2020)]. Fungal functional guild analysis was performed according to Nguyen et al. (2016). R script and input files are available as “Supplementary File 2.” The distribution of fungi was visualized using the Venn Diagram Tool in R (Chen and Boutros, 2011).

### Network Analyses

Matrices describing the presence and absence of fungal taxa in plant samples were assembled. For both twigs and buds separately, these matrices were used to build networks (ignoring site identity as no differences were found between sites, see section “Results”), and they were used to calculate a number of standard network indices (Newman, 2003; Rodríguez-Gironés and Santamaría, 2006; Opsahl et al., 2008); full details are in “Supplementary File 3: Network Analyses.” Specifically, two types of networks were constructed: plant–fungi networks, with individual plants on one layer and endophytic fungi on the other layer, and fungi–fungi networks of fungal taxa in a unique layer (this type is obtained by projecting the plant–fungi matrices onto fungi–fungi symmetrical matrices).

Plant–fungi networks include the interactions between one organ of individual plants and the fungal taxa. These networks were described with (a) a *Connectance* index, describing how much a network is populated by interaction links and measuring the proportion of realized links relative to all possible ones; (b) a *Nestedness Temperature* index, which indicates a pattern in which fungi-rich plant samples host both frequently recorded and rare fungi, while less populated plant samples host frequently recorded fungi.

The fungi–fungi networks describe the interactions within the pool of fungi inhabiting a given sample. To investigate the extent of competitive or facilitative interactions within the fungi pool further, fungi–fungi networks were partitioned into “positive” or “negative” co-occurrence networks of fungal taxa, which measure how often two organisms do or do not occur together. In other words, positive and negative species associations within samples can be described with co-occurrence data and are considered proxies of positive and negative interactions between taxa (Berry and Widder, 2014). The fungi–fungi co-occurrence was calculated with the Raup–Crick similarity index which, as in Chase et al. (2011), evaluates the among-sample taxa occurrence similarity so that it approaches 0 or 1 if taxa are more dissimilar or more similar in their occurrence than expected by random chance; this is tested with 999 null models (in which the probability of selecting species is proportional to the species frequencies). To derive the networks of positive and of negative interactions, pairs of taxa with an occurrence similarity higher than 0.5 were used for the “positive” networks; otherwise, if lower than 0.49, their interactions were included in the “negative” networks (see “Supplementary File 3 and Supplementary Figure 4”). For the fungi–fungi networks, the positive and negative co-occurrence networks were described with indices that can reveal patterns of direct links between fungal taxa and the distribution of facilitative or competitive interactions (Ovaskainen et al., 2010; Berry and Widder, 2014). We included (c) the index of the *Small-world effect*, which measures the possibility of connecting most fungi by a small number of links and is a measure of how close the fungi are to each other (based on their links); (d) the index of the *Rich-club effect*, which describes whether most of the network links occur among the pool of fungi with many links; and (e) the *Assortativity* index, which measures whether fungi link to other fungi having the same number of links (and thus not only whether they have a high number of links as in the Rich-club effect).

The (a–e) indices were tested for significant differences as a function of the plant organ; specifically, the observed difference between a plant organ with a given network index was compared with the distribution of differences resulting from 999 randomly simulated twig networks with the same number of random bud networks (i.e., a network permutation), using a one-tailed test (left or right tail depending on the sign of the difference), as in Biella et al. (2019).

## Results

### Basic Illumina Data Survey

The first analyses revealed four samples with outlying molecular data (i.e., samples with only a few hundred or more than 300k reads). They were removed before further data curation. The final data set contained ca. 730,000 demultiplexed and quality-filtered ITS1 reads from 16 samples with an average of 45,619 reads per sample (range 7,766–122,310). We defined a total of 113 fungal OTUs. Thirty-nine OTUs were found in mountain samples, 58 in samples from the valley, 71 in twigs, and 41 in buds. Thirty-three OTUs were commonly shared among sites and organs (Figure 4B). Common OTUs in valley trees included *Debaryomyces* and unnamed Helotiales and Pleosporales (“Supplementary File 2”/Suppl file 2_input files_OTU97_master_sheet/shared OTUs). Common OTUs in mountain trees included *Ramularia* and *Herpotrichia*.

### Richness Patterns and Community Composition

**FIGURE 2:**
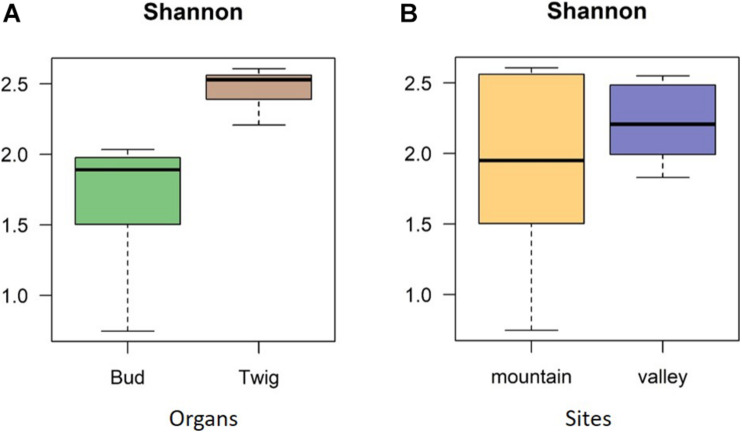
Diversity indices. Shannon diversity indices confirmed mycobiome differences between plant organs **(A)** but not between sites **(B)**. The diversity indices investigated differences with respect to species richness and abundance, as indicated by operational taxonomic units (OTUs).

No significant correlation was found between taxon richness and sequencing depth (Supplementary Figure 1, linear model: *F* < 0.87, *p* = 0.1932) for the whole data set. A significant similarity was found between buds and twigs (*R* = 44%, *p* < 0.01); however, buds hosted only 59.69% of the taxa found in twigs. Taxa accumulation curves adjusted for unequal sample sizes further suggested that twigs hosted a richer community of fungal species than buds (Figure 1B), but when adjustments were made for unequal sample sizes, no difference in species richness was found between valley and mountain sites (Figure 1C). ANOVAs of the multivariate generalized linear model showed (Table 1) that buds and twigs differed significantly (*p* < 0.05, indicated in bold) for all five diversity indices. This substantial difference between the two substrates was consistent between the two sites and across all diversity measurements (Figure 2A and Supplementary Figure 2). On the other hand, the diversity index for mycobiota showed that mountain and valley sites did not differ significantly (Figure 2B). Library size (read number per sample before downsampling of data) had no measurable effect on fungal diversity (Table 2). Consequently, diversity analyses of the downsampled (rarefied) data did not deviate from the above mentioned results (Supplementary Figure 6a and Supplementary Table 1).

**TABLE 1:** Five diversity statistics supporting Figure 2.

	Fisher alpha			Shannon			Hill N1			Hill N2			Hill N3		
					
	Sum of squares	Deviance	*p*	Sum of squares	Deviance	*p*	Sum of squares	Deviance	*p*	Sum of squares	Deviance	*p*	Sum of squares	Deviance	*p*
Buds vs twigs	13.75	32.7	**0.021**	2.46	1.19	**0.036**	151.67	16.38	**0.001**	79.02	12.05	**0.002**	54.72	9.6	**0.002**
Valley vs mountain	2.91	0.78	0.29	0.32	0.16	0.4	4.97	0.39	0.56	2.13	2.32	0.572	1.29	0.22	0.628
Valley buds vs valley twigs	1.36	0.33	0.37	0.46	0.21	0.16	37.65	4.12	**0.007**	21.27	3.11	**0.026**	14.45	2.45	**0.034**
Mountain buds vs mountain twigs	16.07	4.9	**0.03**	2.31	1.19	0.1	129.89	11.27	**0.014**	64.77	9.06	**0.008**	45.41	7.73	**0.015**

*Analyses were based on GLM or multispecies generalised linear models (significance levels, *p* < 0.05 in bold).*

**TABLE 2:** Statistical comparison of differences in network indices between plant organs, based on the analyses of plant–fungi networks and fungi–fungi networks.

Network type	Index	Delta (index_*twig*_ – index_*bud*_)	*p*-Value
Plant–fungi	Connectance	−0.078	0.173
Plant–fungi	Nestedness (T)	9.22	**0.003**
Fungi–fungi	Small-world	0.692	0.423
Fungi–fungi	Rich-club	−0.090	0.779
Fungi–fungi	Assortativity	0.190	**0.010**

*The one-tailed statistical significance was tested with 999 random network permutations, and it is highlighted in bold if *p* < 0.05.*

**FIGURE 3:**
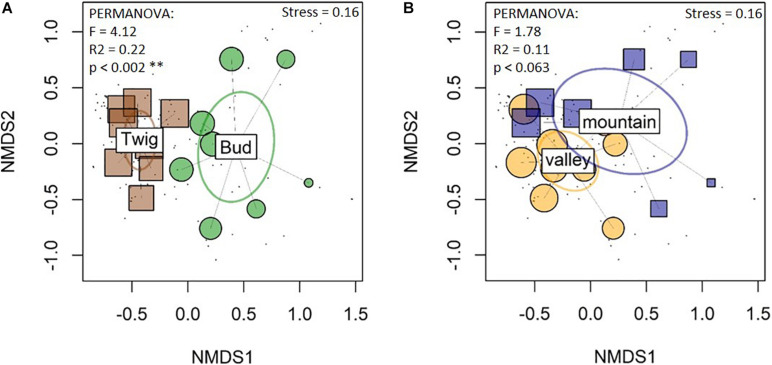
Nonmetric multidimensional scaling (NMDS) of fungal community compositions reveal differences between the organs **(A)** but not between the sites **(B)**. The Bray–Curtis dissimilarity matrix was employed. Box and small circles represent samples. Ellipses represent groups (sites and substrates) of fungal communities: ellipses that overlap represent groups of samples with similar species compositions; bud and twig compositions were significantly different from each other, but there were overlaps between the sites. Twig samples were more similar to each other, i.e., more clustered together, than bud samples.

The fungal community composition (NMDS) supported the diversity measurement and suggested differences between buds and twigs and overlapped between study sites (Figure 3). Community analysis with the rarefied data showed comparable signals (Supplementary Figure 6b).

**FIGURE 4:**
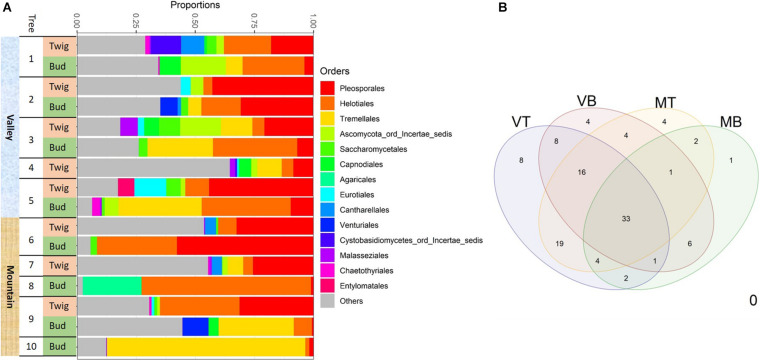
Fungal taxonomic composition showing **(A)** the most 14 abundant taxonomic orders associated with the two sites for each of the two substrates: buds and twigs. More consistent and diverse taxonomic orders were found in twigs but more dynamic orders in buds; this was the case for both valley and mountain sites. Pleosporales was the most abundant order in the twig samples. Helotiales and Tremellales were more common in bud samples. **(B)** Venn diagram of shared taxa in four “sites-organs.” MB, mountain buds; MT, mountain twigs; VB, valley buds; VT, valley twigs.

Determining the taxonomic composition of the mycobiome with use of the UNITE fungal database, we failed to classify approximately 22% of the OTUs (they are hereafter referred to as “unidentified” fungi). Of the 78% of OTUs that could be identified with 97% sequence similarity, two thirds belonged to Ascomycota and one third to Basidiomycota. Abundant taxonomic ascomycota orders included Pleosporales (21% of all OTUs), many of which are saprobes and Helotiales (18% of all OTUs) that are inoperculate discomycetes. The most abundant basidiomycota were Tremellales (16% of all OTUs), of which many are parasites of other fungi. In general, at any growth site, the mycobiome of twigs was taxonomically diverse but also had a rather consistent configuration, whereas the mycobiome of buds appeared more inconsistent in comparison (Figure 4A). Although twigs and buds hosted fungi from all taxonomic orders, Pleosporales were more frequently found in twig samples, whereas taxa belonging to Helotiales and Tremellales were mostly detected in bud samples (Figure 4A).

**FIGURE 5:**
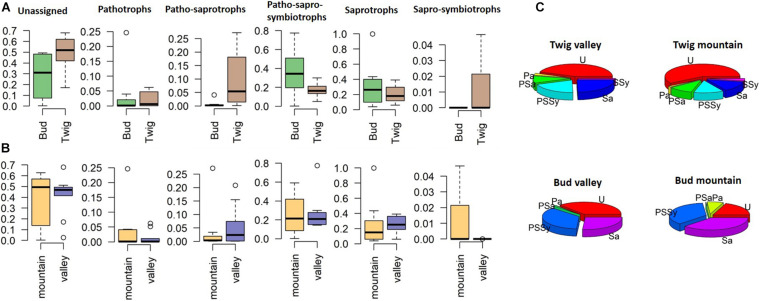
Relative abundance of fungal endophytes divided on the basis of trophic guilds according to FUNGuild (Nguyen et al., 2016). **(A)** Site comparison, **(B)** substrate or organ comparison, and **(C)** pie charts displaying relative abundances per organ and site. U, unassigned; P, pathotrophs; PSa, patho-saprotrophs; PSSy, patho-sapro-symbiotrophs; Sa, saprotrophs; SSy, sapro-symbiotrophs.

Separating the findings into trophic-level membership (irrespective of site), we classified the identified taxa into the following guilds: pathotrophs (P), patho-saprotrophs (PSa), patho-sapro-symbiotrophs (PSSy), saprotrophs (Sa), and sapro-symbiotrophs (SSy) (Figures 5A,B). The representation within the guilds from the twig mycobiomes appeared highly similar between sites, although 44–54% at each site could not be assigned to any guild. PSa were more commonly found associated with twigs than buds, and SSy were only found associated with “mountain” twigs. We were more successful at assigning the mycobiomes of buds to guilds, with only 19 and 37% unassigned taxa (valley and mountain, respectively) compared to twigs where 44 and 59% of the taxa could not assign to a guild (Figure 5C). Although buds had a higher diversity of guild functions than twigs, PSSy dominated at both growth sites. In contrast, twigs were dominated by Sa, indicating a functional difference in the mycobiome depending on the organ.

### Network Variation in Plant Organs

As the first-community analyses and diversity indices of our samples suggested that the organ exerted the greater influence over the endophyte community composition and function, we focused on twig–bud comparisons for the network analyses (Supplementary Figures 3, 4).

The plant–fungi network’s *Nestedness* was significantly higher (*p* = 0.002), and fungi–fungi *Assortativity* was significantly higher (*p* = 0.006) for twigs than buds (Table 2), while the differences between plant organs were small and not significant for the other indices considered (*Connectance*, *Small-world effect*, and *Rich-club effect*). Along with the higher *Nestedness* of twigs, the taxa that were shared between buds and twigs were common in all samples (i.e., 68.05% of taxa were more frequent than the median, across-sample occurrence), while those exclusive of twigs were mostly sample-specific (i.e., 71% of the taxa were less common than the median, across-sample occurrence).

In the fungi–fungi negative co-occurrence networks, only a significantly higher *Rich-club effect* (*p* = 0.04) was detected for the twigs compared to the buds, with small and nonsignificant differences in *Assortativity* and *Small-world effect* (Table 3). The indices for the positive co-occurrence networks did not change significantly between plant organs (Table 3).

**TABLE 3:** Statistical comparison of mycobiome interactions based on the positive and negative co-occurrence networks of fungi–fungi taxa.

Network type	Index	Delta (index_*twig*_ – index_*bud*_)	*p*-Value
Positive interactions	Small-world	0.169	0.566
Positive interactions	Rich-club	−0.005	0.406
Positive interactions	Assortativity	−0.168	0.147
Negative interactions	Small-world	0.078	0.631
Negative interactions	Rich-club	0.133	**0.022**
Negative interactions	Assortativity	−0.025	0.773

*Statistical significance of the difference in indices between plant organs (delta) was tested with 999 random network permutations and is highlighted in bold when *p* < 0.05.*

## Discussion

### Organ Rather Than Growth Site Determines the Beech Microbiome

Until recently, little was known about organ filtering of endophytic communities associated with forest trees, which are constantly subjected to horizontal colonization by fungal inocula from the environment (Witzell and Martín, 2018). Despite some overlap in mycobiota composition, our study identified organ-related differences rather than site effects. Similar mycobiome patterns with a richer twig-associated community compared to that of leaves have previously been reported for beech (Tateno et al., 2015) and for three oak species (*Quercus* spp.) (Ragazzi et al., 2003). In a comparison of gray alder (*Alnus incana*) and hazel (*Corylus avellana*) (Küngas et al., 2020), further concluded that tree organs are the main determinants of fungal community structure, whereas the effect of host species and locality appears less important. In addition, tree health may also play a role (Berg et al., 2017). It has, for example, been suggested that defense barriers are broken down in unhealthy trees, paving the way for saprotrophic fungi. This has also been demonstrated for Pedunculate Oak (*Quercus robur*) by Agostinelli et al. (2018), who found that xylem was a more selective substrate for endophytes compared to bark and, furthermore, that high vitality of trees was associated with reduced habitat quality in relation to wood-associated endophytes. However, detection methods vary between studies and this will affect their outcome (Kowalski and Kehr, 1995; Giordano et al., 2013; Siddique et al., 2017). Studies of beech using NGS techniques do, indeed, suggest that locality can influence mycobiome diversity in leaves, as do leaf age and leaf biochemistry (Siddique and Unterseher, 2016; Unterseher et al., 2016; Siddique et al., 2017); moreover, these studies suggest large temporal and spatial effects on endophyte community composition and dynamics in beech trees, which could also be true for other forest trees.

Fungal biodiversity in this study was lower and less complex than reported for Unterseher et al. (2016), although the plants belonged to the same experimental entity and were planted at the same time under the same conditions. One possible reason for this difference could be that leaves and not buds/twigs were the focus of the previous study. Furthermore, the plants for this study might have been habituated to their new environment (Supplementary Figure 2). According to our daily temperature and humidity measurements (data not shown), the mountain site was covered with snow much longer than the valley. The fungi in the buds from the harsher mountain site may therefore have been less protected compared to fungi in both buds and twigs in the valley environment.

### Endophyte Occurrence in Buds

Bud endophytes were detected using high-throughput sequencing techniques, and surprisingly, bud samples shared almost half of their fungal taxa with twigs, indicating that unburst buds were indeed not free of endophytes. This hypothesis of endophyte-free, undeveloped organs was proposed by Toti et al. (1993) who, based on culturing methods, concluded that spring leaf fragments of Ash (*F. excelsior*) achieved endophyte growth in only 5% of the samples, which, it was argued, could indicate a germ-free environment in the bud followed by rapid colonization by microfungi right after bud burst. In contrast, endophytic fungi isolated from pine tissue cultures originated from buds indicated that fungi are localized to meristematic and scale tissues of the buds (Pirttilä et al., 2003). Our study resembled the study by Chen (2012) who also reported a profound presence of endophytes in New Zealand Ash buds. Our results support the suggestion that it may, indeed, be hard for fungal inoculates to penetrate the bud scales of beech and establish within a bud before leaf break, but due to modern detection methods, our study also captured a more comprehensive picture of the bud mycobiome, which could be the result of early colonization or early insect transportation (Sieber and Hugentobler, 1987; Halmschlager et al., 1993; Kowalski and Kehr, 1995; Scholtysik et al., 2013). However, the possibility cannot be dismissed that buds are systemically infested from the already colonized twig that bears them (Pirttilä et al., 2003). As buds break and leaves emerge and get older, spore deposition accumulates with an increased chance of successful colonization of the leaf (Unterseher et al., 2007; Jumpponen and Jones, 2010). Fungal community members in the overwintering twig may proliferate and spread to adjacent unburst buds with physiological and biochemical features that filter the inocula (Bahnweg et al., 2005; Hashizume et al., 2008; Scholtysik et al., 2013).

### Network Analyses Suggest Competitive Interactions in the Mycobiome

Network analyses of both plant–fungi and fungi–fungi interactions can reveal important patterns of community structure and competitive exclusion. Plant–fungi network analyses suggested similarity between the networks associated with the two organs. However, further subgroup analyses revealed an increase in *Nestedness* of twigs as compared to indices for buds, possibly indicating that the variety of environmental conditions consistent with fungal coexistence was higher in twigs than in buds. Burns, 2007 suggested that a low degree of *Nestedness* among early colonizers of epiphytes could be due to generalist colonizers, which could also be the case for our buds, which were characterized by low *Nestedness* and colonization by mainly ubiquitous endophytes. In addition, an increase in twigs’ *Assortativity* when compared to buds indicates that fungi were increasingly interacting with others of similar link rank (i.e., among fungi with a similar number of interactions) (Newman, 2003). We found a higher *Rich-club* effect in the negative co-occurrence networks of the twigs than the buds. This later result shows that, in twigs, the pairwise fungi–fungi competition occurs mostly within the pool of taxa with many interaction links (Opsahl et al., 2008), suggesting an increased magnitude of pairwise competition happening within the pool of the highly connected fungi. Therefore, first, the higher rate of interactions and of competition occurring between similarly connected fungi in twigs than in buds could indicate that competitive exclusion is an emerging mechanism in the transition from buds to twigs (Goldberg and Barton, 1992; Halley et al., 1994). Second, the higher *Nestedness* of plant–fungi for buds is compatible with a scenario where buds are early stages of an ecological succession in which few generalist, tolerant species occur (Grime, 1973); this may occur more frequently in areas prone to random environmental changes, which could be driven by quick shifting or seasonal conditions.

Taxa associated with twigs were the most diverse and consistent, and some taxa were shared with those found in buds, suggesting that those fungi extend their succession from one substrate to another (Küngas et al., 2020). In this study, we identified five major guilds (P, PSa, PSSy, Sa, and SSy), but not the sixth guild “symbiotroph” available in the FUNGuild reference database (Nguyen et al., 2016). Twig samples tended to be richer in pathogenic and wood-decaying fungi. The relative abundance of pathotrops and symbiotrophs differed from the leaf-inhabiting endophyte communities found in a previous study (Siddique et al., 2017), supporting the suggestion of organ-based differences. Because of insufficient fungal data in FUNGuild, it has been seen in the present study that a large proportion of fungi, especially twigs, cannot be assigned to a Guild.

### Temporal Effects

Endophytic communities change over seasons (Osono, 2008; Albrectsen et al., 2010; Mishra et al., 2012) and with leaf age (Siddique and Unterseher, 2016), even over short periods of time (Peršoh et al., 2013). The five most abundant fungal taxa from our study of early season organs were *Alternaria*, *Cryptococcus*, *Hymenoscyphus*, *Herpotrichia*, and *Tetracladium*, respectively. In pine, twigs were rich in Pleosporales fungi, e.g., *Alternaria* (Bußkamp et al., 2020). *Hymenoscyphus* and *Lachnum* were only found in buds whereas *Candida*, *Chaetomium*, *Itersonilia*, *Setomelanomma*, *Sterigmatomyces*, and *Sistotrema* were only found in twigs. Potentially, as buds generated substantial quantities of foam during sample processing, they may contain chemicals that behave differently during the analysis protocol compared to other organ types.

The lack of significant differences between the two sites at two different altitudes contradicts the current hypothesis of habitat specificity and is in contrast to findings from earlier studies of the same sites and habitats but at different times, in which site differences were found for leaf-inhabiting fungal assemblages (Siddique and Unterseher, 2016; Unterseher et al., 2016; Siddique et al., 2017). One explanation is that the taxa associated with early buds and twigs are not comparable with taxa of the fully grown leaves because each plant organ has its own unique history, suggesting that the mycobiome may initially be a result of random colonization events. In previous studies, leaves had 900 OTUs, 600 OTUs, and 597 OTUs, respectively (Siddique and Unterseher, 2016; Unterseher et al., 2016; Siddique et al., 2017), compared to 113 OTUs in the current study. Thus, as the mycobiome changes over the seasons (Albrectsen et al., 2010; Mishra et al., 2012; Juybari et al., 2019; Karlsson et al., 2020), older leaves may be richer in endophytes, and thus, the fungal community and composition found in the spring samples (current study) may differ from autumn samples previously reported in Siddique and Unterseher (2016), samples that were also collected from another stand of beech trees.

## Conclusion

The current study provides a snapshot of the fungal communities of European beech collected during one sampling event in spring; it covers fungal endophytes in twigs and buds that were identified by high-throughput sequencing. Analyses of fungal networks and their ecological guilds provided insight into how mycobiome structure in the subjected tissues is influenced by functional roles.

With the price of high-throughput sequencing going down, more sites could be compared and the effect of other traits such as season, organ filtering, and plant age could be detailed. Our understanding is still limited about the functional roles endophytic fungal species may potentially play inside their host. Consequently, any attempt to extrapolate functional roles across plant species or environmental conditions will inevitably include uncertainties. With fungal endophytes having the potential to reveal insights about tree health and site effects, metagenomic analyses such as those presented here promise to be strong tools that could be used in any forest monitoring for example for management or conversation purposes.

## Data Availability Statement

The datasets presented in this study can be found in online repositories. The names of the repository/repositories and accession number(s) can be found below: https://www.ncbi.nlm.nih.gov/bioproject/PRJNA689109, accession numbers: SRX9767996, SRX9767995, and SRX9767994

## Author Contributions

AS and MU organized the project and conducted the experiments and lab works. AS worked on the primary acquisition of the data and bioinformatics analysis, and wrote the manuscript’s first draft. Downstream analysis: AS did the diversity, composition, and taxonomic analysis and PB did the taxa screening and network analysis. AS, PB, and BA wrote the final manuscript. AS and PB contributed to writing equally. MU supervised the project. All authors read, edited, and approved the final manuscript.

## Conflict of Interest

The authors declare that the research was conducted in the absence of any commercial or financial relationships that could be construed as a potential conflict of interest.

## Footnotes

1 www.r-project.org, last accessed November 2020.
