# Supplementary material for: Mycobiomes of Young Beech Trees Are Distinguished by Organ Rather Than by Habitat, and Community Analyses Suggest Competitive Interactions Among Twig Fungi
Source: Front Microbiol. 2021 Apr 15;12:646302. doi: 10.3389/fmicb.2021.646302 (PMC8086555; doi:10.3389/fmicb.2021.646302)
Supplement: Supplementary file 3 [file Data_Sheet_3.docx]

***Supplementary file 3.***
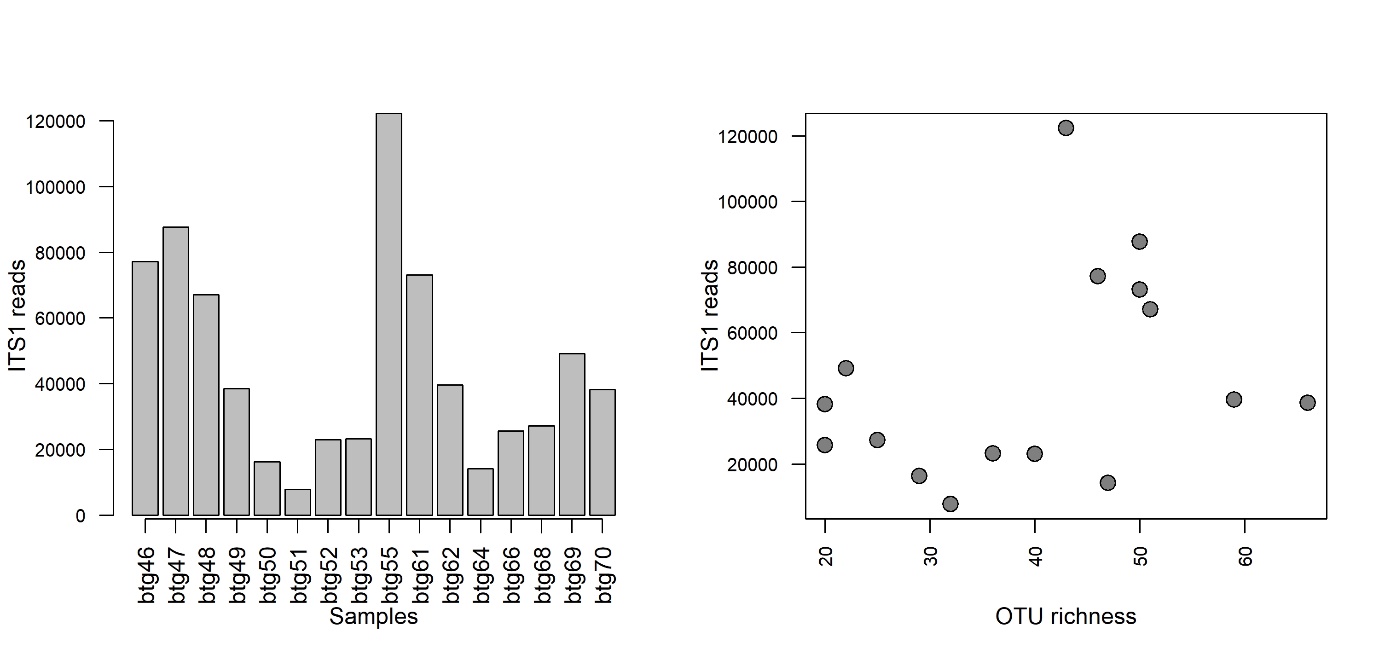


Figure S1. Sequencing effort is not correlated with OTU richness.


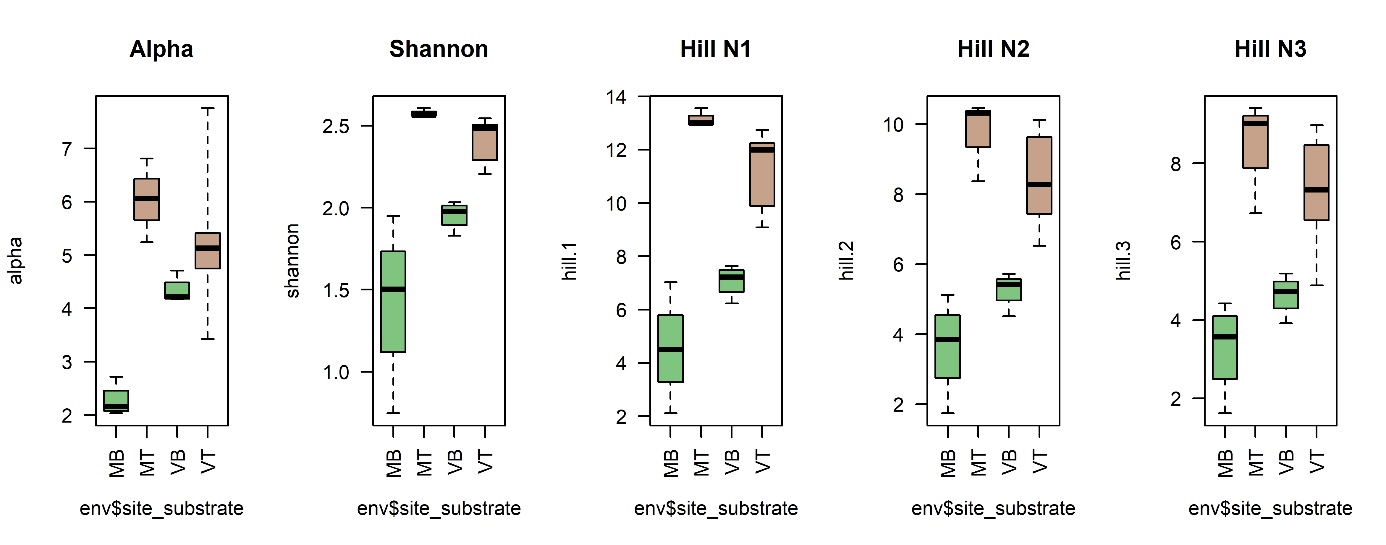


## Figure S2. Five diversity indices for the sites and substrates. All fungal diversity indices showed significantly higher values for the twigs than the buds at both sites. MB= Mountain-Buds, MT= Mountain-Twigs, VB= Valley-Buds, VT= Valley-Twigs.

**Network analyses**

The networks did not include those OTUs that were represented only by a very low number of reads in a given sample (typically being in the tail of a per-sample statistical distribution of reads), based on the suggestions of Serrao *et al.* (2018) for environmental DNA data and applied to DNA metabarcoding data by Biella *et al.* (2019). In other words, we used ROC curves for calculating reliable thresholds for the exclusion of unreliable OTUs (i.e. with few DNA sequencing reads), instead of arbitrarily selecting fixed cut-off values. Specifically, for each sample, we assigned a variable coded as “negative” if an OTU had no DNA sequencing reads, and “positive” if an OTU had DNA sequencing reads >0; a generalised linear regression with a quasipoisson distribution (that is an overdispersed Poisson distribution) was performed for each sample so that we could estimate the distribution of reads related to positives and negatives; the values predicted by the regressions were fitted in the *pROC* package (Robin *et al.* 2011) in the R environment to calculate the per-sample cut-off threshold and thus to identify which OTUs could be excluded from each sample.

For each tissue type, plant–fungi, and fungi–fungi networks were constructed. The latter were used for creating co-occurrence networks of fungal taxa based on the Raup-Crick similarity index among plant samples of the same tissue (see main text). To build these co-occurrence networks, the interactions between fungal taxon pairs with occurrence similarity higher than 0.5 were used for the “positive” networks; otherwise, when the pairwise similarity was lower than 0.49, the interactions between fungal taxa were included in the “negative” networks. If the similarity was 0, then NAs were included in order to control for the ambiguity of total dissimilarity (were two given taxa never co-occurred or were they not found because of the sampling). The magnitude of the similarity (or of the dissimilarity) was included as an interaction weighting in the positive (or negative) networks.

The indices chosen for describing network structure were as follows: (a) Connectance: calculated as in Bersier *et al.* (2002), which can vary from 0 to a maximum of 1; (b) Nestedness Temperature index: calculated as in Rodríguez‐Gironés & Santamaría (2006), which ranges from 0 (highly nested) to 100 (no nestedness); (c) Small-world index: calculated as in Newman (2003); (d) the Rich-club index: calculated as in Opsahl *et al.* (2008) and here it includes the nodes with a link number higher than the across-nodes mean; (e) the Assortativity index: calculated as in Newman (2003). Indices (a) to (b) were calculated from plant × fungi matrices, and these were projected as fungi–fungi symmetrical matrices with entries being the fungi taxa found on the same plant sample for calculating indices (c) to (e).

**References**

Bersier, L.-F., Banašek-Richter, C., and Cattin, M.-F. (2002). Quantitative descriptors of food-web matrices. *Ecology* 83, 2394–2407.

Biella, P., Tommasi, N., Akter, A., Guzzetti, L., Klecka, J., Sandionigi, A., et al. (2019). Foraging strategies are maintained despite workforce reduction: A multidisciplinary survey on the pollen collected by a social pollinator. *PLOS ONE* 14. doi:[10.1371/journal.pone.0224037](https://doi.org/10.1371/journal.pone.0224037).

Newman, M. (2003). The structure and function of complex networks. *SIAM REVIEW* 45, 167–256. doi:[10.1137/S003614450342480](https://doi.org/10.1137/S003614450342480).

Opsahl, T., Colizza, V., Panzarasa, P., and Ramasco, J. J. (2008). Prominence and control: the weighted rich-club effect. *Physical review letters* 101, 168702.

Robin, X., Turck, N., Hainard, A., Tiberti, N., Lisacek, F., Sanchez, J.C. and Müller, M., (2011). pROC: an open-source package for R and S+ to analyze and compare ROC curves. *BMC bioinformatics* 12, 1–8.

Rodriguez-Girones, M., and Santamaria, L. (2006). A new algorithm to calculate the nestedness temperature of presence-absence matrices. *Journal of Biogeography* 33, 924–935. doi:[10.1111/j.1365-2699.2006.01444.x](https://doi.org/10.1111/j.1365-2699.2006.01444.x).

Serrao, N.R., Reid, S.M. and Wilson, C.C., 2018. Establishing detection thresholds for environmental DNA using receiver operator characteristic (ROC) curves. *Conservation Genetics Resources* 10, 555–562.


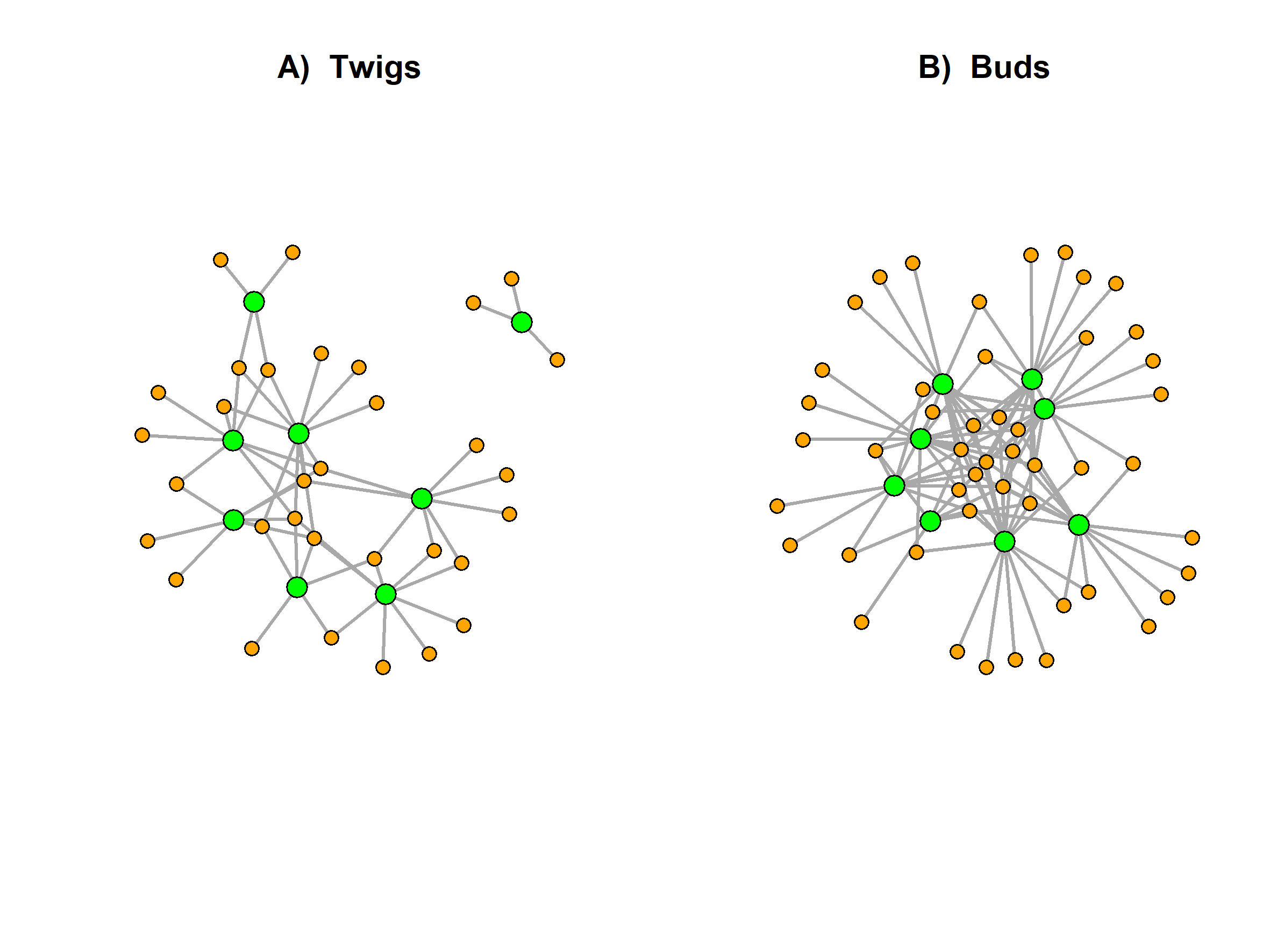


Figure S3. Plant–fungi interaction network for (A) twigs and (B) buds; green denotes plant individuals and orange indicates fungi species.


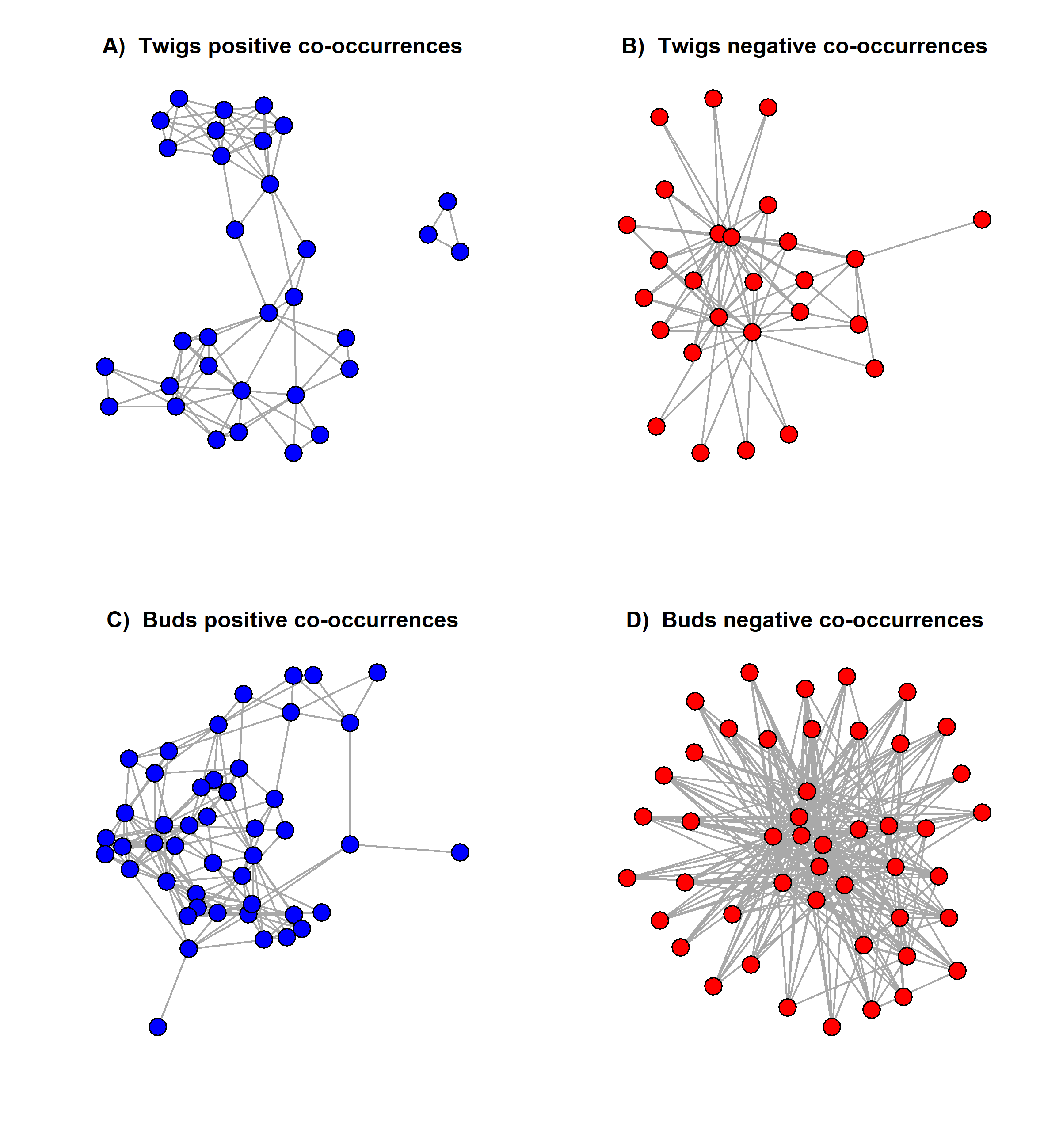


Figure S4. Networks of fungi–fungi interactions for (A) positive co-occurrence in twigs, (b) negative co-occurrence in twigs; and (C) positive co-occurrence in buds and (D) negative co-occurrence in buds.


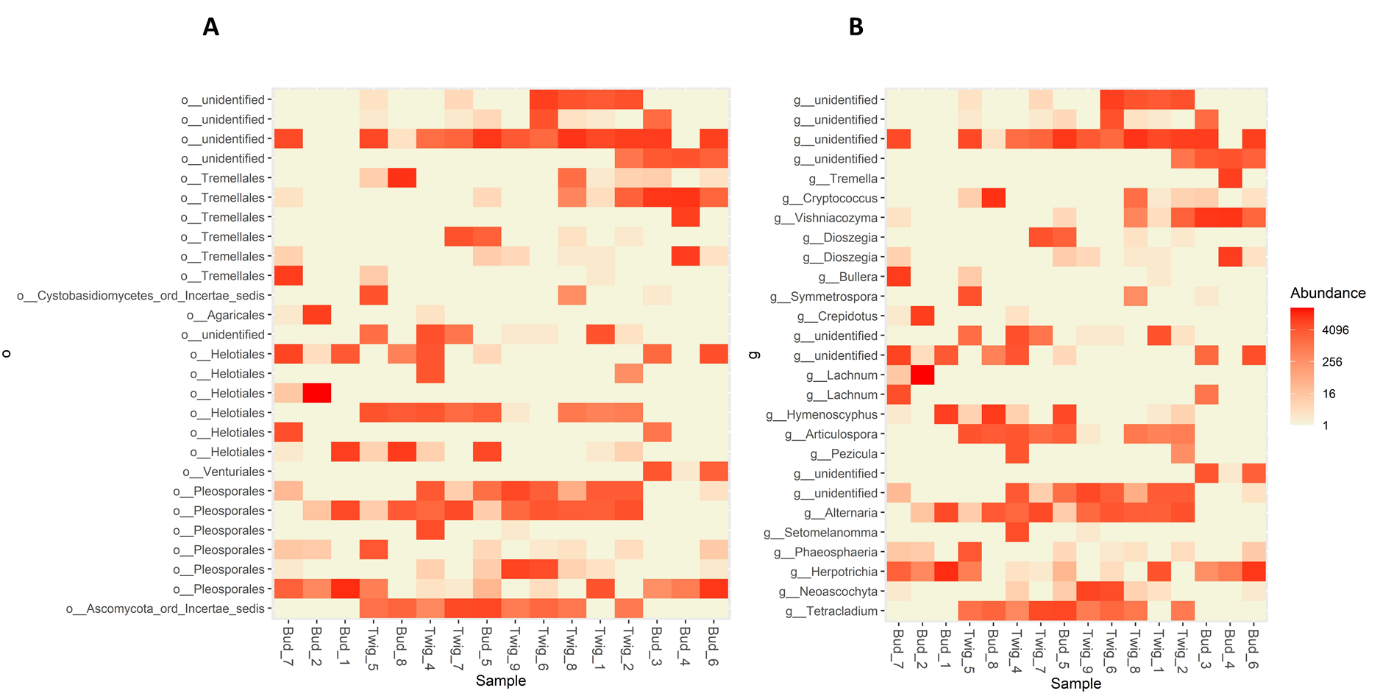


Figure S5. Heatmap of the most abundant orders (A) and genera (B) in the mycobiome of beech tissues. The left half of each heatmap shows community composition for buds, which are characterised by sample-specific patterns of a few abundant genera. Genera from twigs (right half) show higher levels of co-occurrence and a less pronounced abundance of single genera


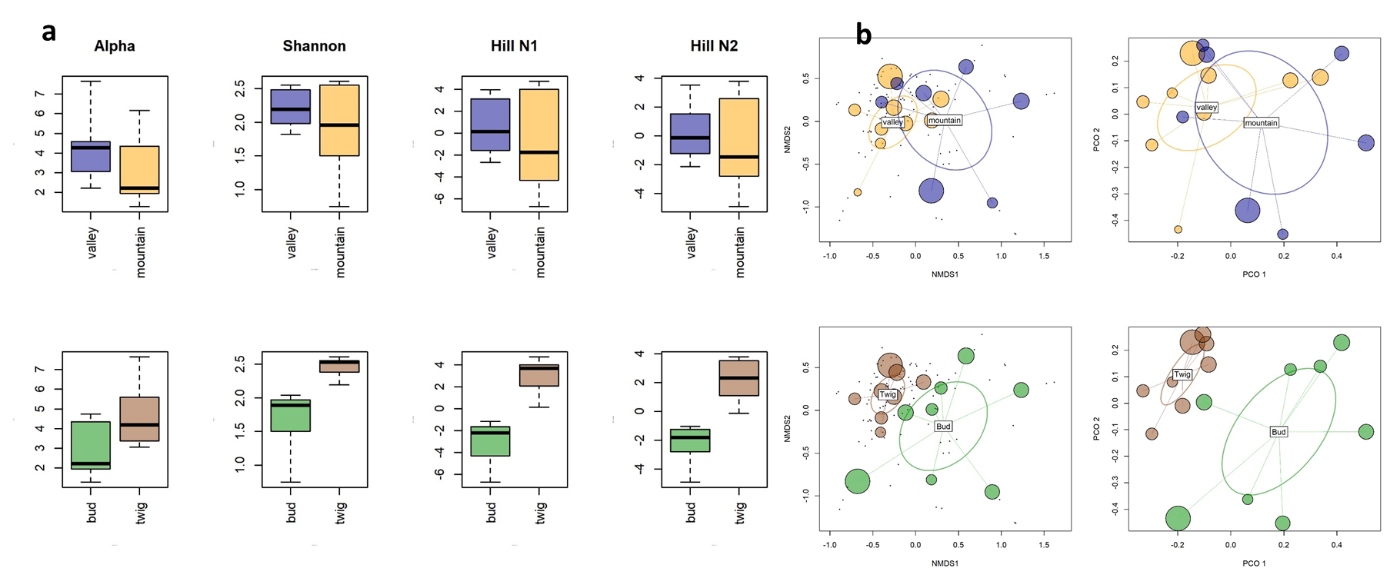


Figure S6: Diversity and compositional analysis using rarefied data. [a] shows five diversity indices (upper panel between the two sites and lower panel between the organs). [b] shows Non-metric multidimensional scaling (NMDS) and Principle coordinate analysis (PCO) between the sites and between the organs (upper panel: between the two sites; lower panel: between the organs)

**Statistics**

Table S1. Five diversity indices supporting figure 2. Analyses were based on GLM or multispecies generalised linear models (significance levels, P < 0.05 in bold).

> summary(lm(rowSums(spec)~rowSums(spec > 0))) #no correlation!

Call:

lm(formula = rowSums(spec) ~ rowSums(spec > 0))

Residuals:

Min 1Q Median 3Q Max

-37052 -21519 -5945 17764 74193

Coefficients:

Estimate Std. Error t value Pr(>|t|)

(Intercept) 15070.3 23635.4 0.638 0.534

rowSums(spec > 0) 768.5 562.2 1.367 0.193

Residual standard error: 30800 on 14 degrees of freedom

Multiple R-squared: 0.1178, Adjusted R-squared: 0.05475

F-statistic: 1.869 on 1 and 14 DF, p-value: 0.1932

> summary(lm(rowSums(spec.mountain)~rowSums(spec.mountain > 0))) #no correlation!

Call:

lm(formula = rowSums(spec.mountain) ~ rowSums(spec.mountain >

0))

Residuals:

btg61 btg62 btg64 btg66 btg68 btg69 btg70

31041 -4747 -27119 -8768 -8467 14234 3826

Coefficients:

Estimate Std. Error t value Pr(>|t|)

(Intercept) 29320 19028 1.541 0.184

rowSums(spec.mountain > 0) 254 501 0.507 0.634

Residual standard error: 20430 on 5 degrees of freedom

Multiple R-squared: 0.0489, Adjusted R-squared: -0.1413

F-statistic: 0.2571 on 1 and 5 DF, p-value: 0.6337

> summary(lm(rowSums(spec.valley)~rowSums(spec.valley > 0))) #no correlation!

Call:

lm(formula = rowSums(spec.valley) ~ rowSums(spec.valley > 0))

Residuals:

Min 1Q Median 3Q Max

-43829 -23365 -14861 22499 71795

Coefficients:

Estimate Std. Error t value Pr(>|t|)

(Intercept) -9018 53671 -0.168 0.871

rowSums(spec.valley > 0) 1384 1194 1.159 0.284

Residual standard error: 38170 on 7 degrees of freedom

Multiple R-squared: 0.1611, Adjusted R-squared: 0.04128

F-statistic: 1.344 on 1 and 7 DF, p-value: 0.2843

> summary(lm(rowSums(spec.twig)~rowSums(spec.twig > 0))) #no correlation!

Call:

lm(formula = rowSums(spec.twig) ~ rowSums(spec.twig > 0))

Residuals:

Min 1Q Median 3Q Max

-36003 -22640 -1292 22825 35808

Coefficients:

Estimate Std. Error t value Pr(>|t|)

(Intercept) 23808.1 53550.9 0.445 0.672

rowSums(spec.twig > 0) 560.3 1055.2 0.531 0.614

Residual standard error: 29910 on 6 degrees of freedom

Multiple R-squared: 0.04488, Adjusted R-squared: -0.1143

F-statistic: 0.282 on 1 and 6 DF, p-value: 0.6145

> summary(lm(rowSums(spec.bud)~rowSums(spec.bud > 0))) #no correlation!

Call:

lm(formula = rowSums(spec.bud) ~ rowSums(spec.bud > 0))

Residuals:

Min 1Q Median 3Q Max

-35125 -27181 -2388 15115 63109

Coefficients:

Estimate Std. Error t value Pr(>|t|)

(Intercept) -4555 44893 -0.101 0.922

rowSums(spec.bud > 0) 1483 1449 1.023 0.346

Residual standard error: 35420 on 6 degrees of freedom

Multiple R-squared: 0.1486, Adjusted R-squared: 0.006652

F-statistic: 1.047 on 1 and 6 DF, p-value: 0.3457
