# Supplementary figures and images for: Mycobiomes of Young Beech Trees Are Distinguished by Organ Rather Than by Habitat, and Community Analyses Suggest Competitive Interactions Among Twig Fungi
Source: Front Microbiol. 2021 Apr 15;12:646302. doi: 10.3389/fmicb.2021.646302 (PMC8086555; doi:10.3389/fmicb.2021.646302)

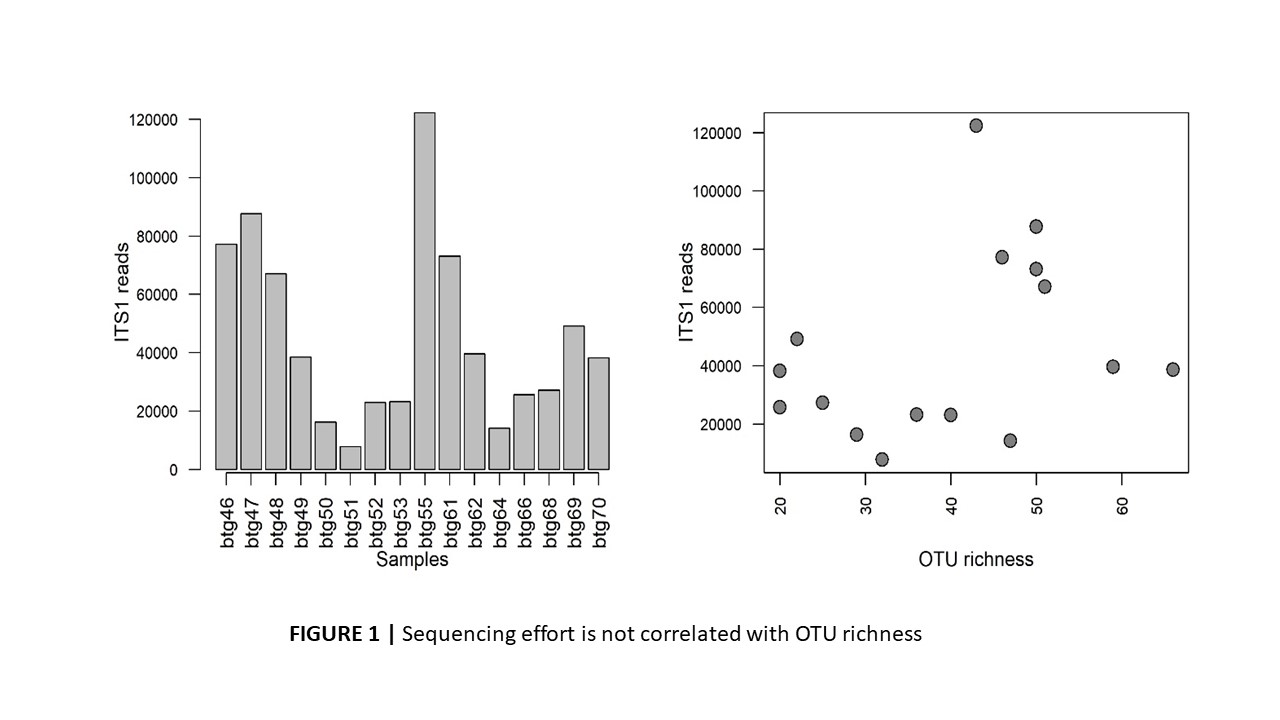

Supplement: Supplementary file 4 [file Image_1.jpg]

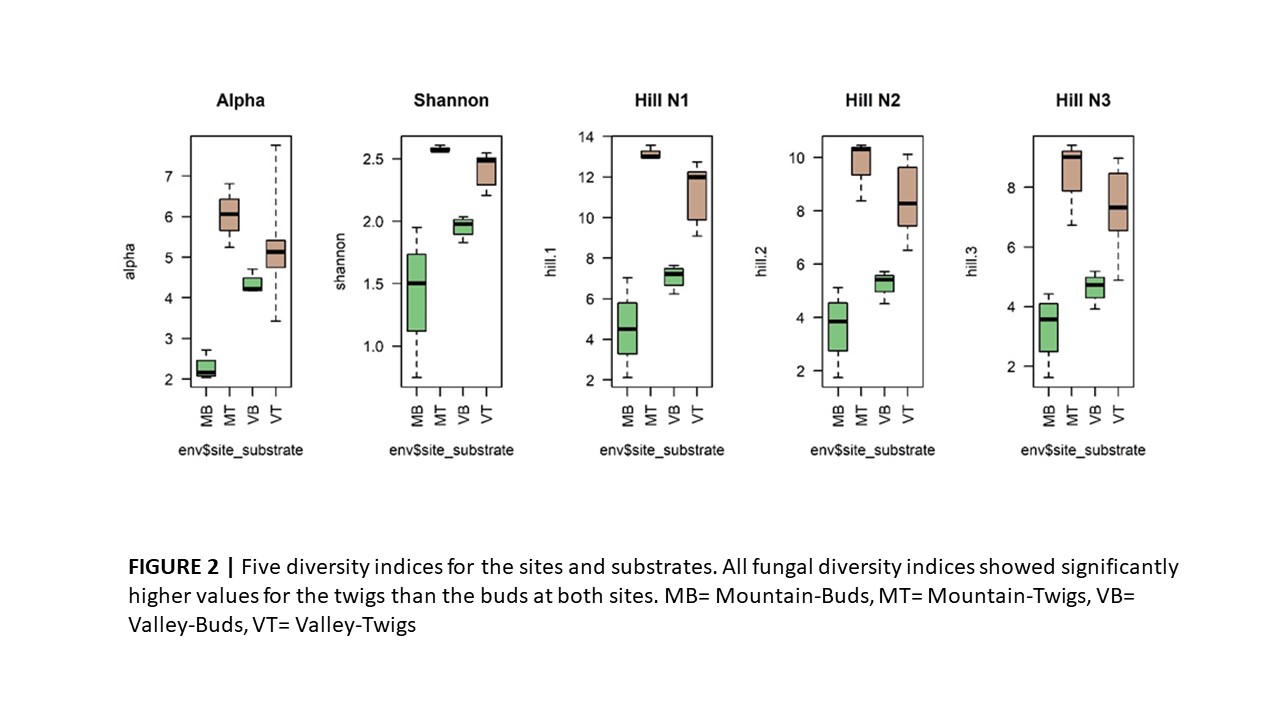

Supplement: Supplementary file 5 [file Image_2.jpg]

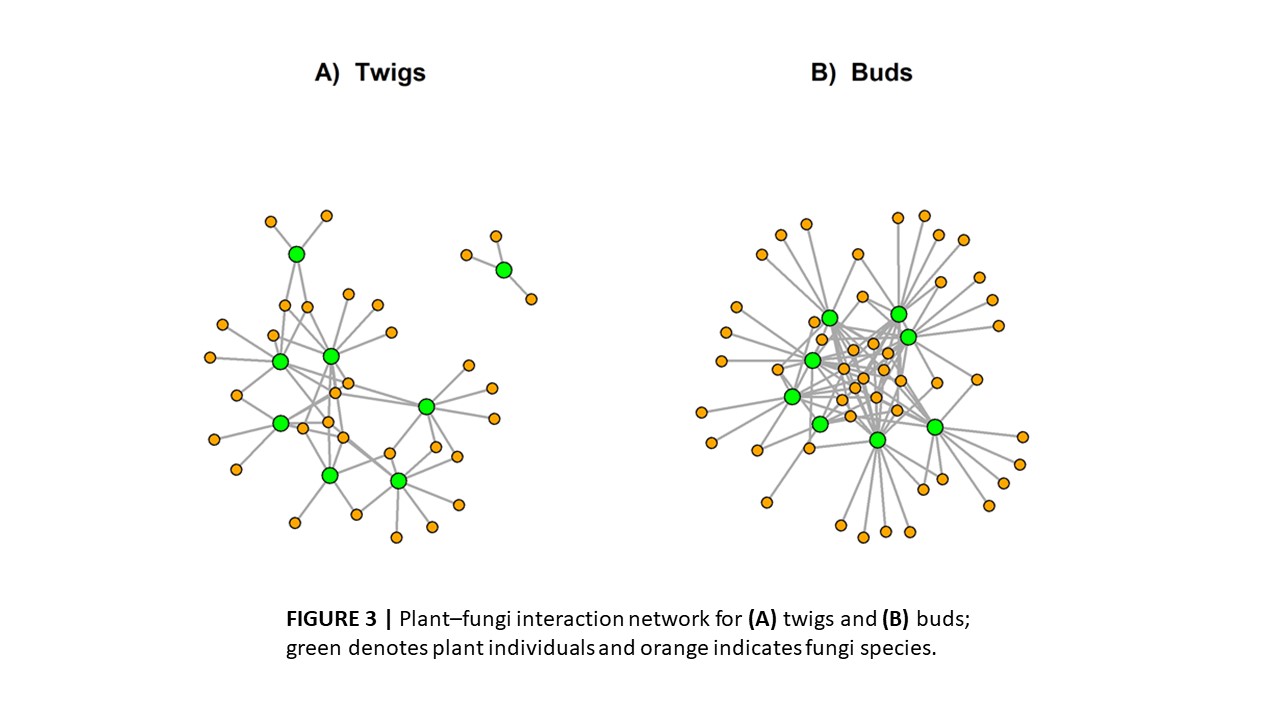

Supplement: Supplementary file 6 [file Image_3.jpg]

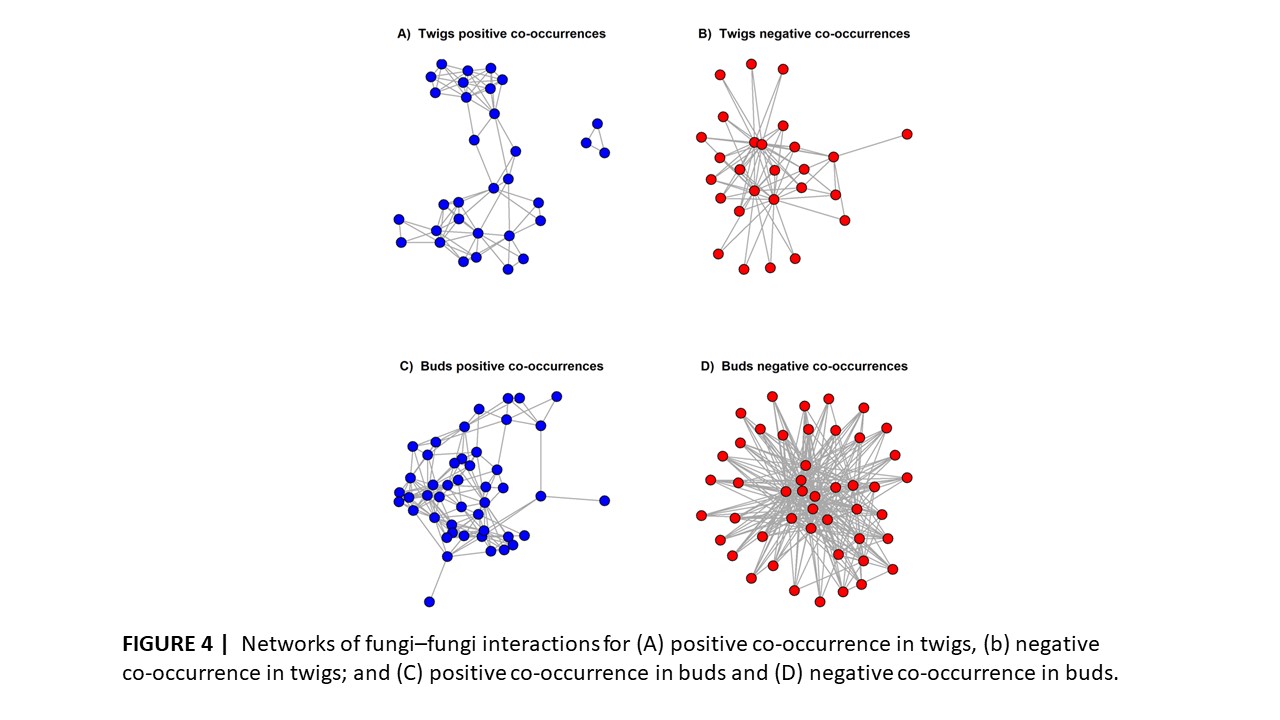

Supplement: Supplementary file 7 [file Image_4.jpg]

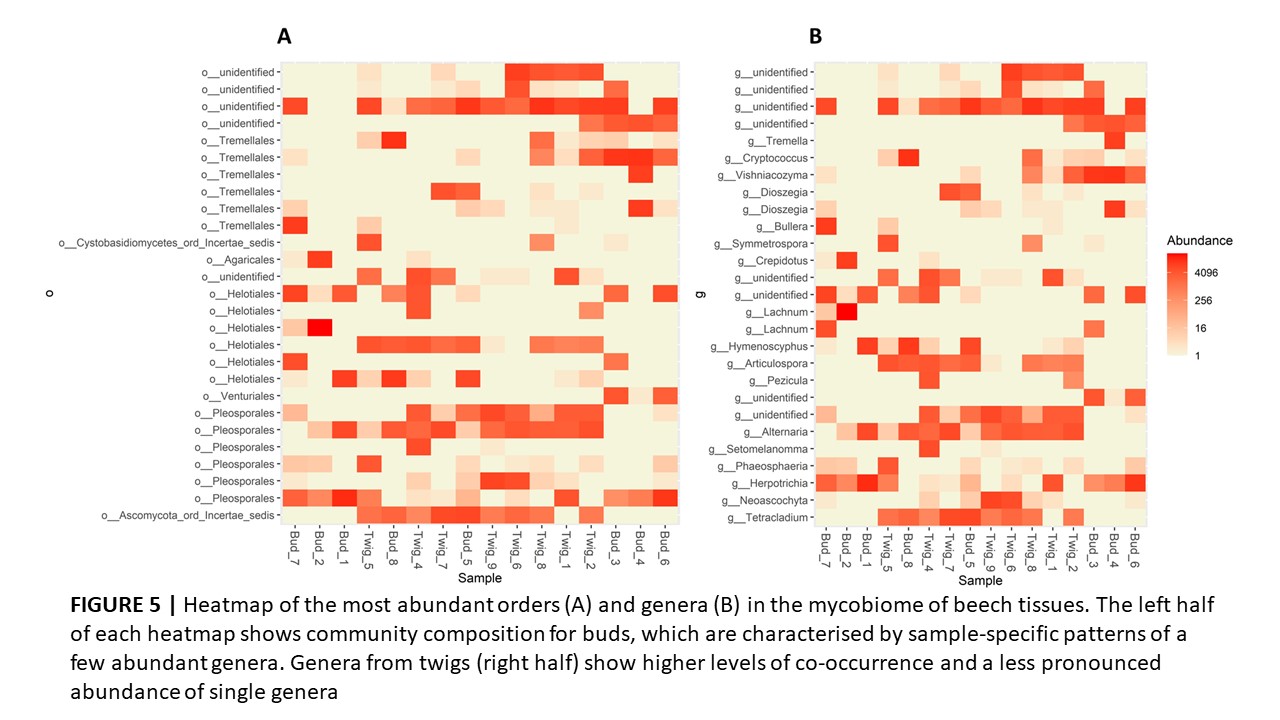

Supplement: Supplementary file 8 [file Image_5.jpg]

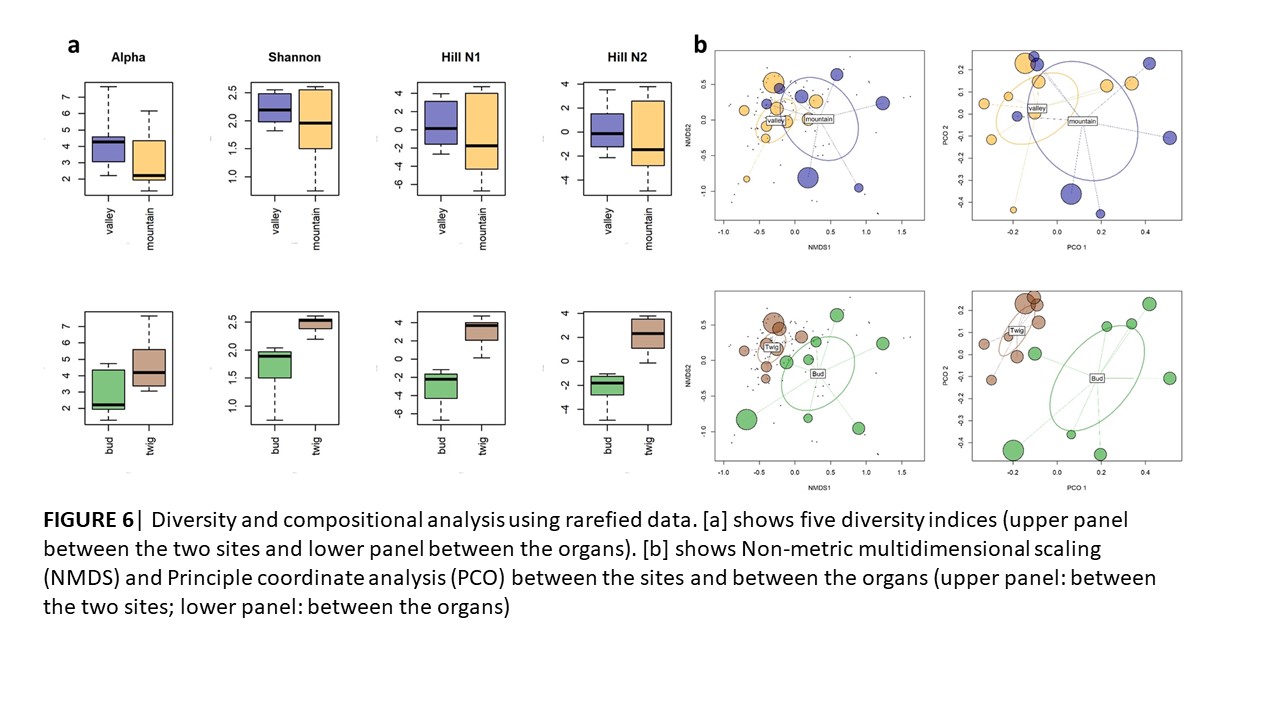

Supplement: Supplementary file 9 [file Image_6.jpg]
