# Supplementary material for: Mycobiomes of Young Beech Trees Are Distinguished by Organ Rather Than by Habitat, and Community Analyses Suggest Competitive Interactions Among Twig Fungi
Source: Front Microbiol. 2021 Apr 15;12:646302. doi: 10.3389/fmicb.2021.646302 (PMC8086555; doi:10.3389/fmicb.2021.646302)
Supplement: Supplementary file 10 [file Table_1.pdf]

TABLE 1 | Five diversity indices supporting figure 2. Analyses were based on GLM or multispecies generalised linear models (significance levels, $p < 0.05$ in bold).

	Fisher alpha			Shannon			Hill N1			Hill N2			Hill N3		
	**Sum of squares**	**Deviance**	***p***	**Sum of squares**	**Deviance**	***p***	**Sum of squares**	**Deviance**	***p***	**Sum of squares**	**Deviance**	***p***	**Sum of squares**	**Deviance**	***p***
Buds vs Twigs	13.75	3.72	**0.021**	2.46	1.19	**0.036**	151.67	16.38	**0.001**	79.02	12.05	**0.002**	54.72	9.6	**0.002**
Valley vs Mountain	2.91	0.78	0.29	0.32	0.16	0.4	4.97	0.39	0.56	2.13	0.32	0.572	1.29	0.22	0.628
Valley-buds vs Valley-twigs	1.36	0.33	0.37	0.46	0.21	0.16	37.65	4.12	**0.007**	21.27	3.11	**0.026**	14.54	2.45	**0.034**
Mountain-buds vs Mountain-twigs	16.07	4.9	**0.03**	2.31	1.19	0.1	129.89	11.27	**0.014**	64.77	9.06	**0.008**	45.41	7.73	**0.015**
